# Supplementary material for: Disruption of Transmembrane Phosphatidylserine Asymmetry by HIV-1 Incorporated SERINC5 Is Not Responsible for Virus Restriction
Source: Biomolecules. 2024 May 10;14(5):570. doi: 10.3390/biom14050570 (PMC11118262; doi:10.3390/biom14050570)
Supplement: Supplementary file 1 [file biomolecules-14-00570-s001.zip › biomolecules-2953684-supplementary.pdf]

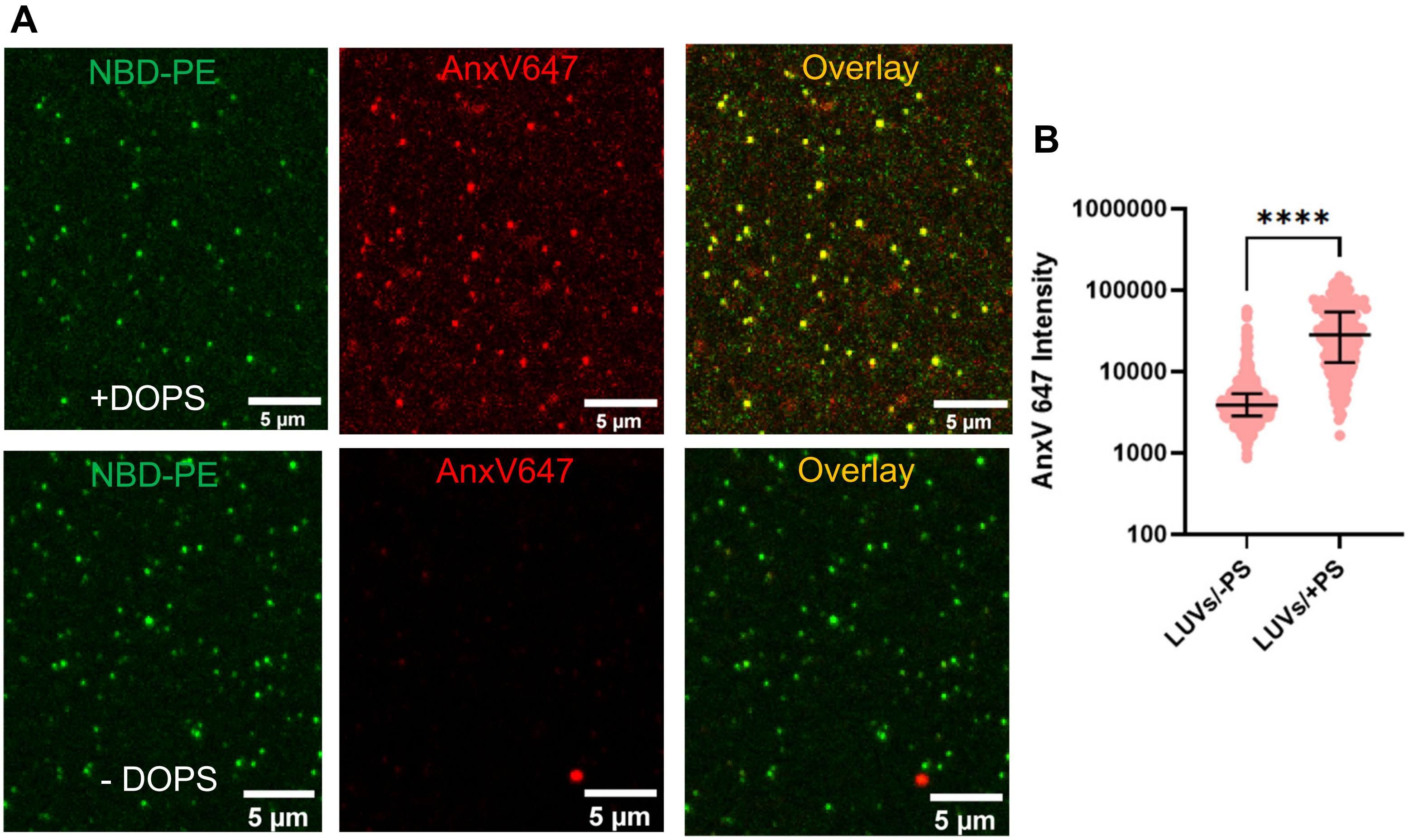

Figure S1. Detection of PS on surface-immobilized liposomes. (A) Representative confocal images of surface-immobilized large unilamellar vesicles with 10mol% DOPS (upper panel) and without DOPS (10 mol% DOPC- lower panel). Liposomes containing 0.3mol% NBD-PE (green) as a fiducial were subsequently stained with AnxV A647 (red). (B) Representative scatterplot showing mean AnxV intensities of single liposome puncta from multiple FOVs in each sample (N>100). Statistical analysis was performed using Kolmogorov–Smirnov test. ****, $p < 0.0001$.

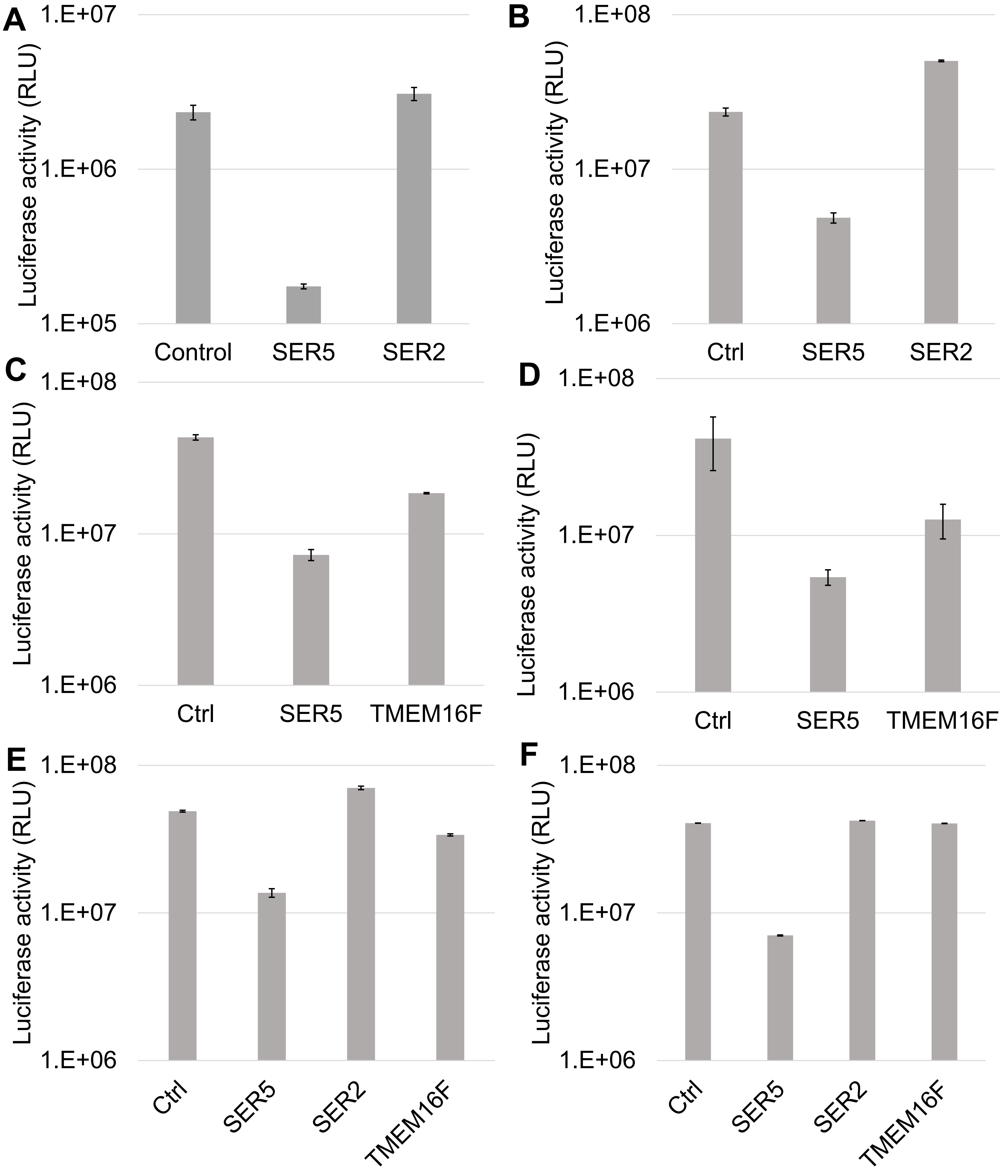

Figure S2. Functional characterization of pseudovirus preparations. (A-F) Representative p24 normalized luminescence readings from luciferase reporter assay plotted for independent biological replicates. Related to main Fig. 1F.

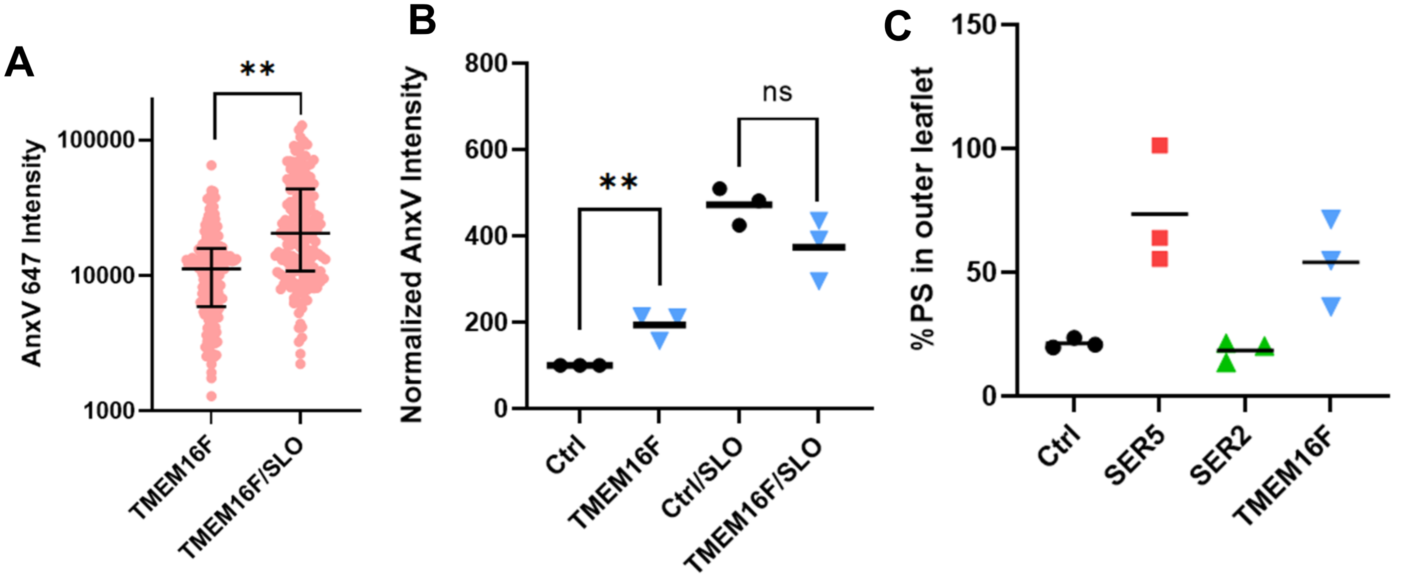

Figure S3. SLO permeabilization of TMEM16F viruses. (A) Representative scatterplot showing AnxV intensity distribution of TMEM16F samples before and after permeabilization by SLO. (B) Raw AnxV intensity values for 3 independent biological replicates were normalized to the Ctrl of the respective samples and plotted. (C) %PS in the outer leaflet of the viruses in panel (A) was calculated and plotted. Ctrl, SER5 and SER2 samples from Figure 3C were plotted alongside TMEM16F to aid visual clarity. The symbols indicate values from each replicate, and the horizontal line denotes the mean value. Statistical analysis for (A) was performed using Kolmogorov–Smirnov test. Statistical analysis for (B) and (C) were performed using student's t-test. n.s., $p > 0.05$; *, $0.05 > p > 0.01$; **, $0.01 > p > 0.001$.

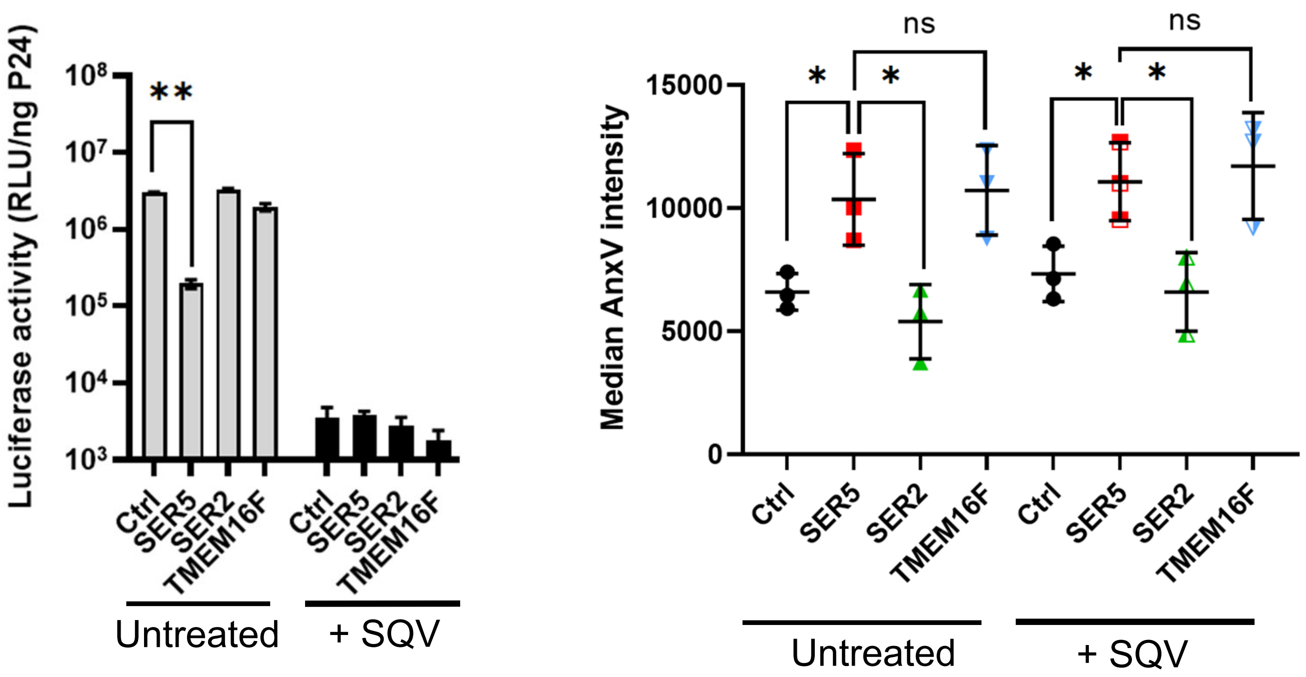

Figure S4. Effects of Saquinavir treatment on pseudoviral infectivity and AnxV staining. (A) Representative luciferase reporter assay showing the functional impact of SQV (black bars) treatment on infectivity of pseudoviruses. Control viruses (untreated) are shown in grey bars.(B) Median AnxV intensity values of pseudoviruses made in presence of SQV, compared to Untreated samples obtained from 3 biological replicates are plotted and analyzed. Symbols denote values from each biological replicate. The middle horizontal lines and error bars represent mean and S.D., respectively. n.s., $p > 0.05$; *, $0.05 > p > 0.01$; **, $0.01 > p > 0.001$. Note that $p>0.05$ wasn't shown for (A) to aid visual clarity.

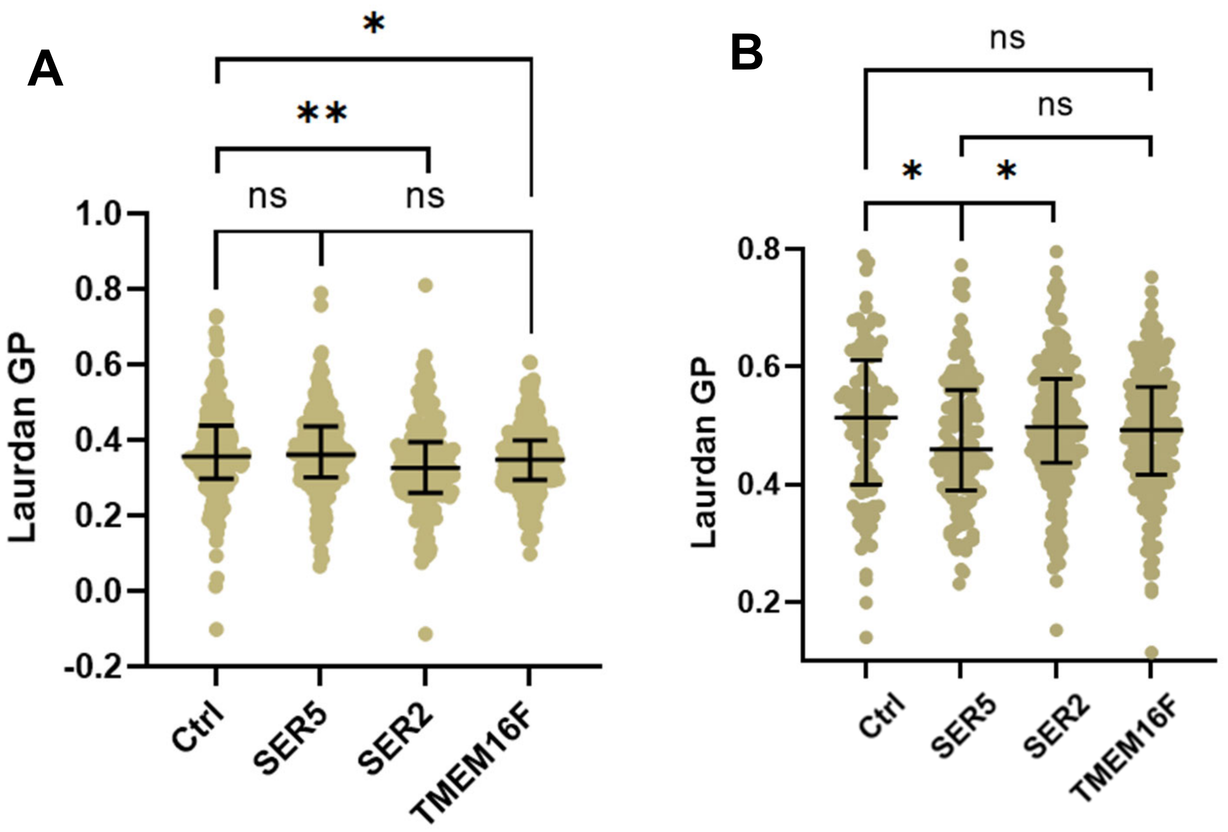

Figure S5. Lipid order measurements on viruses. (A and B) Representative Laurdan GP values from single-viruses containing mRFP-Vpr are plotted for HIV-1 pseudoviruses lacking (Ctrl) or containing SER5, SER2 or TMEM16F. (A) and (B) represents two independent biological preparations. The middle horizontal line represents the median value, and the error bars represent the interquartile range. n.s., $p > 0.05$; **, $0.01 > p > 0.001$.

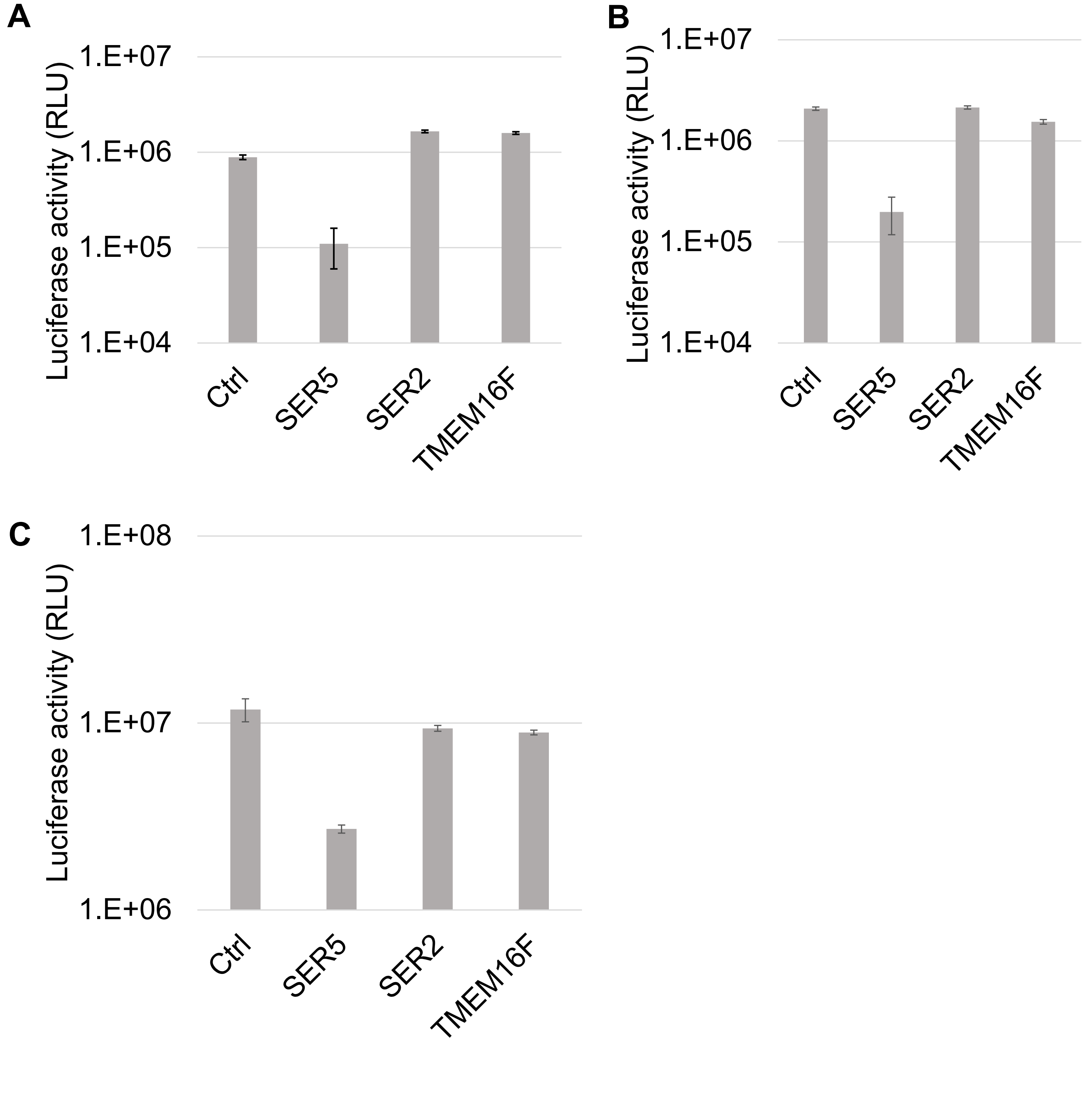

Figure S6. Functional characterization of pseudovirus preparations produced from ΔCDC50a cells. (A)-(C) Representative p24 normalized luminescence readings from luciferase reporter assay plotted for 3 independent biological replicates. Related to main Fig. 5G.

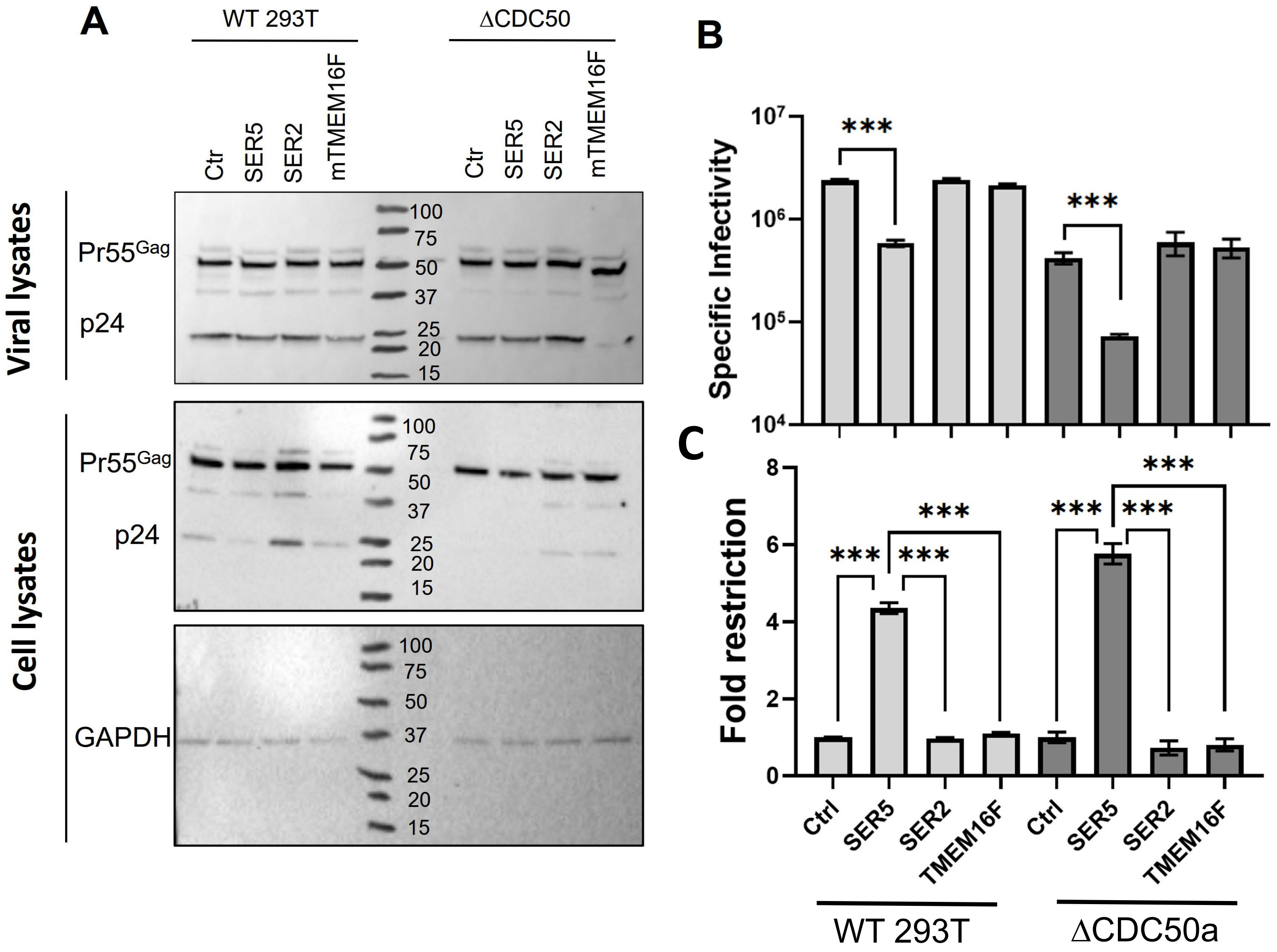

Figure S7. Western blot characterization of viruses and producer cells. (A) Representative Western blots show comparable levels of viral protein expression in cell lysates (middle panel) and Gag processing in viral lysates (top panel) between WT 293T cells and ΔCDC50a 293T cells. Bottom panel shows GAPDH bands in cell lysates obtained from both 293T and ΔCDC50a virus producer cells. (B) Representative p24 normalized luminescence readings from luciferase reporter assay for the viral preparations analyzed in A. Specific infectivity was plotted as luciferase signal per ng of p24 virus for each condition. (C) Fold infectivity reduction (Fold restriction) relative to Ctrl for both WT 293T cells and ΔCDC50a 293T is plotted. The bars and the error bars represent the mean value and SD, respectively. Statistical analysis was performed using student's t-test. ***, $p < 0.001$. Note that $p>0.05$ is not shown in (B) and (C) to aid visual clarity.

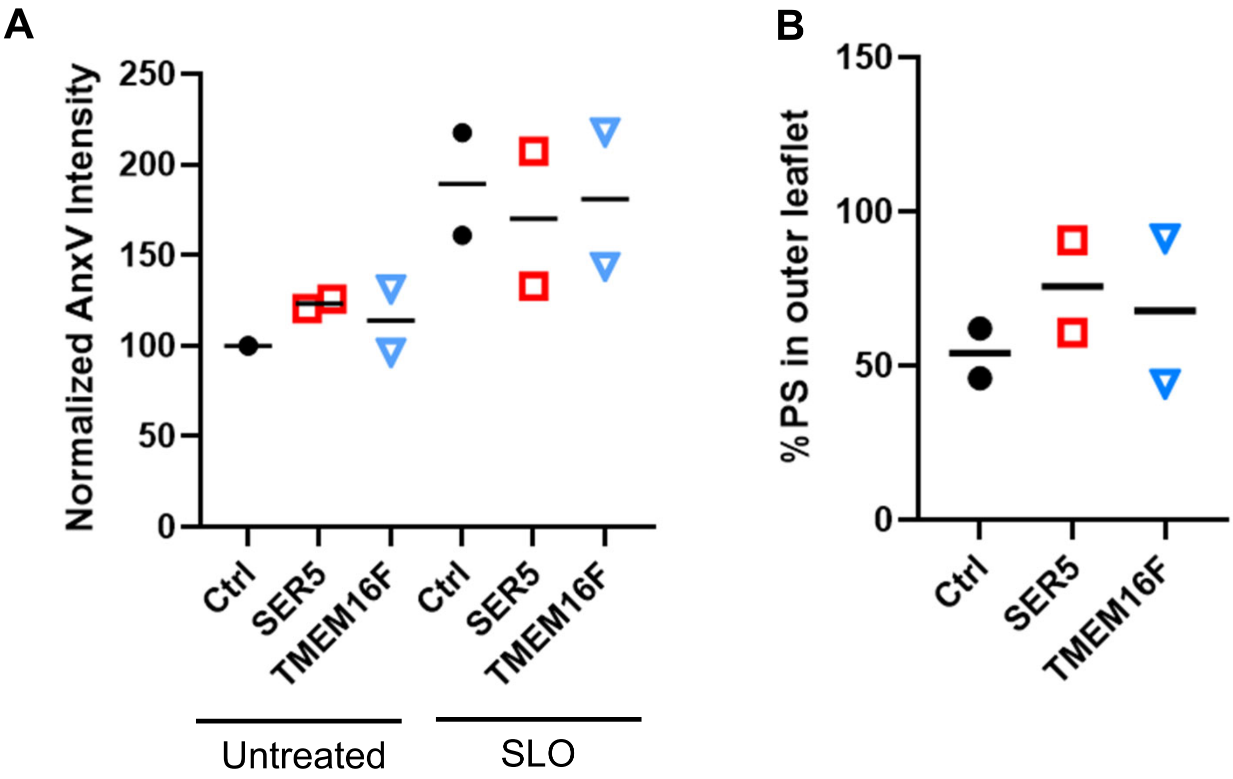

Figure S8. SLO permeabilization of viruses produced from ΔCDC50a cells. (A) Median AnxV intensity values in from each independent biological replicate (N=2) were normalized to the Ctrl of the respective samples and plotted. (B) %PS in the outer leaflet of the viruses in panel (A) was calculated and plotted. The symbols indicate values from each replicate, and the horizontal line denotes the mean value. Statistical analysis was performed with student's t-test. $p>0.05$ were not plotted to aid visual clarity.

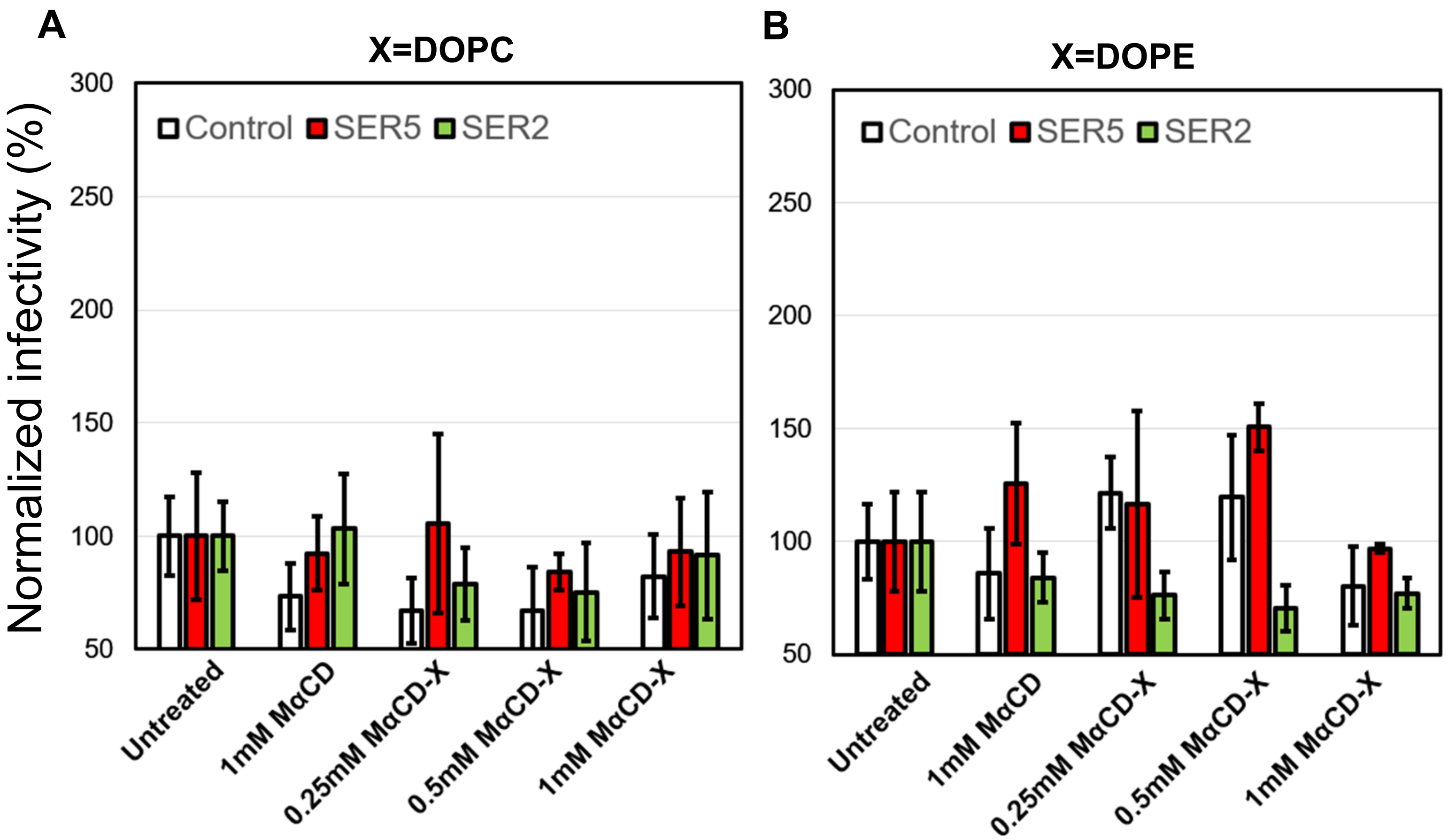

Figure S9. Functional effects of treating viruses with MαCD-DOPC (A) and MαCD-DOPE (B) complexes. Infectivity data obtained from 3 independent biological replicates were normalized to the respective untreated samples and plotted. The bars and the error bars represent the mean value and S.D., respectively. Statistical analysis was performed with student's t-test. $p>0.05$ were not plotted to aid visual clarity.

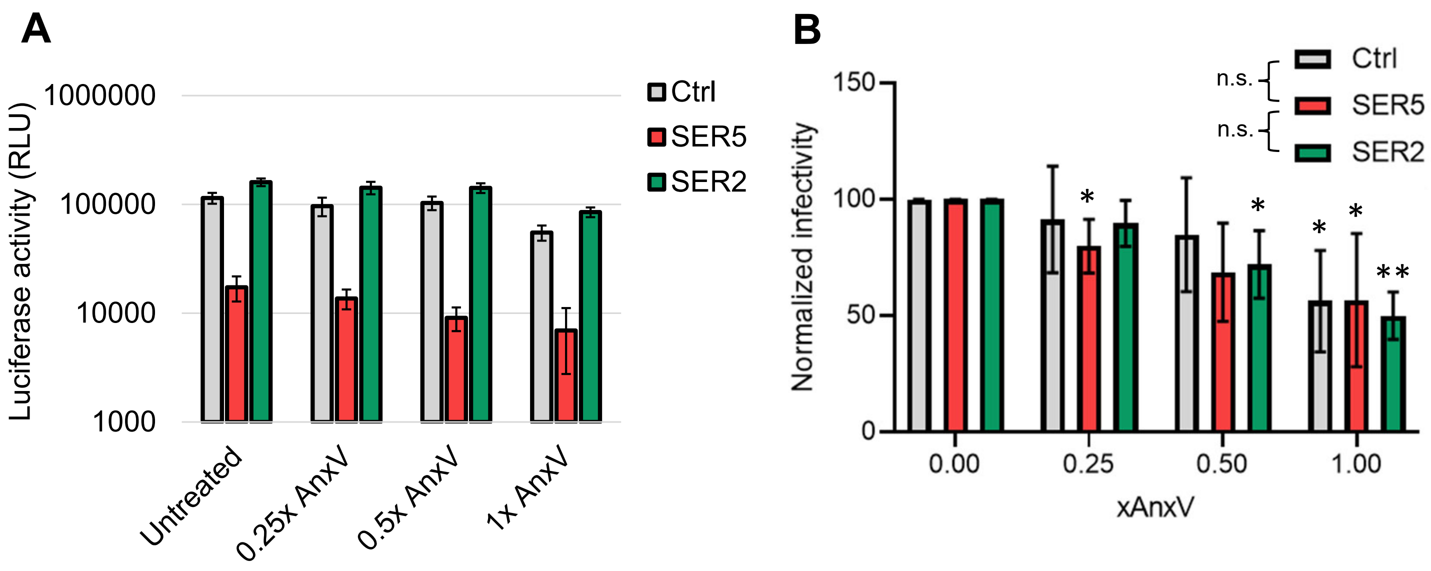

Figure S10. Effects of pre-binding AnxV on pseudoviral infectivity. (A) Raw luminescence readings from Luciferase reporter assay plotted as a function of different AnxV concentrations. (B) Normalized infectivity (relative to untreated) values from 3 biological replicates were plotted as a function of different AnxV. The bars and the error bars represent the mean value and S.D., respectively. Statistical analysis was performed with student's t-test. Individual data points were compared to their respective untreated controls. n.s., $p > 0.05$; *, $0.05 > p > 0.01$; **, $0.01 > p > 0.001$. $p>0.05$ were not plotted above the bars to aid visual clarity.
